# Incidence and Burden of Endometriosis Among U.S. Active Component Service Women, 2017–2024

**Published:** 2026-04-01

**Authors:** Jessica H. Murray, Sithembile L. Mabila

**Affiliations:** Epidemiology and Analysis Branch, Armed Forces Health Surveillance Division, Public Health Directorate, Defense Health Agency, Silver Spring, MD

## Abstract

Endometriosis is a complex gynecological condition affecting nearly 10% of reproductive-aged women. This report updates a 2017 *MSMR* report of gynecological conditions, including endometriosis, from 2012 through 2016 among U.S. active component service women. The current report utilized medical encounter data from 2017 through 2024 to assess the incidence of endometriosis and its health care burden among U.S. active component service women. Factors related to co-occurring gynecological conditions, deployment, parity, and contraceptive use were also examined. Crude incidence rates and incidence rate ratios with 95% confidence intervals were calculated. The overall crude rate of endometriosis was 32.8 cases per 10,000 person-years and increased approximately 42.0% from 2017 to 2024. Incidence rates increased with age and were higher among nulliparous and never-deployed service women. Additionally, obese and underweight service women had higher incidence rates. Menorrhagia was the most common co-occurring condition, with oral birth control the most common form of contraceptive among incident cases. Identification of at-risk service women may help formulate targeted policies for earlier diagnosis to improve both quality of life and military readiness.

**What are the new findings?**

Incidence of endometriosis increased during the surveillance period, from 28.7 cases per 10,000 person-years in 2017 to 40.7 cases per 10,000 person-years in 2024, coincident with a general increase of medical encounters for endometriosis, from 2,740 medical encounters in 2017 to 3,864 medical encounters in 2024. Service women who were older, obese or underweight, nulliparous, and never deployed had higher incidence rates.

**What is the impact on readiness and force health protection?**

Endometriosis is associated with a multitude of symptoms and co-occurring gynecological conditions that can negatively affect daily life, military readiness, and deployability. These findings enable the Military Health System to better identify at-risk service women and formulate policies for earlier diagnosis and treatment, improving quality of life in addition to preserving military readiness.

Endometriosis is a complex gynecological condition in which endometrial-like tissue grows outside the uterus. ^1^ Symptoms of endometriosis include dysmenorrhea, dyspareunia, severe pelvic pain, and infertility, although some women present as asymptomatic. ^2^ Approximately 10% of reproductive-age women are affected by this condition ^2 , 3^ ; however, true prevalence may be under-estimated due to differing case criteria and diagnostic biases in many studies. ^3 , 4^ Furthermore, prevalence estimates may be influenced by the use of surgical visualization to obtain definitive diagnosis in addition to delays in surgical diagnosis from symptom onset, which average 7 years. ^5^ Socio-demographic characteristics, reproductive history, contraception use, personal habits, and body characteristics have been evaluated as potential risk and protective factors for endometriosis, but the literature is inconsistent. ^2 , 6^

Women account for approximately 17% of U.S. active component service members, of whom approximately 98% are of reproductive age (ages <20–49 years). ^7^ From 2012 to 2016, endometriosis affected an average of 1,113 U.S. active component service women (ACSW) annually, accounting for an annual average of 2,470 medical encounters and 195 bed days each year. ^8^ Those findings suggest a significant loss of duty time for ACSW due to endometriosis, as well as a heavy burden on the Medical Health System (MHS). Older ACSW, those in the Army, and non-Hispanic Black service women were reported to have higher incidences of endometriosis than their respective counterparts. ^8^ Co-occurring conditions, including menorrhagia, polycystic ovarian syndrome (PCOS), and uterine fibroids, may also put service women at a higher risk for endometriosis. ^8^

Risk and protective factors for endometriosis are inconsistent in the literature. ^2 , 9^ Among protective factors, an inverse relationship between body mass index (BMI) and endometriosis has been demonstrated. ^2 , 6^ Furthermore, women of greater parity have also shown reduced risk of endometriosis when compared to nulliparous women and women with lower numbers of pregnancies. ^10 , 11^ In addition, current or recent oral contraceptive use (risk ratio 0.4, 95% CI 0.2, 0.7) reduced risk of endometriosis by 60% compared to never-users; however, protective effects of oral contraceptives dissipate among former users. ^2^ While current or recent intrauterine device (IUD) use has demonstrated reduced risk of endometriosis, other studies have shown no association between IUDs and endometriosis diagnosis. ^2^

From 2012 to 2016, the incidence of endometriosis among ACSW was reported as 30.8 cases per 10,000 person-years (p-yrs). ^8^ This rate is notably higher than a 2006–2015 U.S. population-based study that reported an average incidence rate (IR) of 24.3 cases per 10,000 p-yrs. ^12^ While health care accessibility and affordability provided by the MHS could explain the higher incidence of endometriosis in the military population, when compared to the civilian population, there is no clear reason why military women are more likely to suffer from endometriosis. Military service-related effects—of deployment, mental health, and reproductive health—could offer other explanations for this anomaly, however. ^9 , 13^

Combat ACSW must be mission ready to deploy; endometriosis and symptomology may hinder this ability, however. Furthermore, combat-related deployments have been linked to physical and mental health issues. ^14 , 15^ Female veterans of Operation Enduring Freedom (OEF) and Operation Iraqi Freedom (OIF) with mental health issues were more likely to receive a diagnosis of endometriosis compared to female veterans without mental health issues; differences were not found to be due to demographics, service characteristics, or primary care. ^9^ Negative coping strategies due to personal- and deployment-related stressors may influence reproductive health risks such as unintended pregnancy or deprioritized reproductive health care. ^13^ Additionally, women who served in OEF, OIF, and Operation New Dawn (OND) with deployments longer than 9 months were more likely to be diagnosed with infertility, ^15^ a condition linked to endometriosis. ^2^ A recent report of ACSW of reproductive potential found incidence of infertility at 77.5 cases per 10,000 p-yrs ^16^ ; infertility affects 30.0–50.0% of women with endometriosis. ^17^ These effects not only raise concerns about reproductive health but force readiness overall.

Previous reports on gynecological disorders among ACSW showed decreased overall annual incidence for all conditions evaluated—with the exceptions of endometriosis and uterine fibroids; IRs for endometriosis and uterine fibroids remained stable. ^8^ While updated incidence and burden research for uterine fibroids exists, ^18^ there is a lack of literature singularly focused on endometriosis among ACSW.

While several factors have been consistently observed as protective against diagnosis for endometriosis, those findings have not been widely reported for military populations. The purpose of this study was to assess the incidence of endometriosis diagnosis and its health care burden among U.S. ACSW. Due to the lag in time between onset and diagnosis, true incidence of endometriosis could be determined; therefore, all references to incidence in this report refer to diagnosis incidence rather than onset of symptom incidence. Compared to prior research on this population, ^8^ relationships between incident endometriosis and deployment, BMI, parity, and contraceptive use were explored, as these factors may influence endometriosis diagnoses. Co-occurring gynecological conditions and endometriosis were also analyzed, similar to previous reporting, ^8^ to help better understand the reproductive health of ACSW.

## Methods

### Study population

The study population consisted of all ACSW ages 17–62 years in any branch of service of the U.S. Armed Forces, excluding the Coast Guard, from January 1, 2017 through December 31, 2024. Demographic, deployment, and inpatient and ambulatory care medical encounter records were obtained from the Defense Medical Surveillance System (DMSS); deployment data, only available through December 2022, were analyzed to determine numbers and lengths of deployments at any time prior to incident dates. Demographic variables included age, service branch, racial or ethnic group, rank, marital status, BMI, and occupation. BMI was obtained through the Defense Centers for Public Health–Portsmouth (DCPH-P) MHS Data Repository (MDR) and Periodic Health Assessments (PHAs). BMI records were excluded if height was less than or equal to 1 meter (m); height greater than or equal to 2.5 m; weight less than or equal to 20 kilograms (kg); weight greater than or equal to 180 kg; or obtained during pregnancy. The BMI record closest to the incident date was used for cases, while the BMI record closest to the start of a service record was used for the remaining population.

International Classification of Diseases, 9th and 10th revisions, Clinical Modification (ICD-9-CM / ICD-10-CM) codes were used to determine endometriosis diagnoses and co-occurring gynecological conditions, including menorrhagia, PCOS, uterine fibroids, and infertility **(Table 1)** . In addition to ICD-9-CM / ICD-10-CM codes, Procedure Coding System (ICD-9-PCS / ICD-10-PCS) codes and Current Procedural Terminology (CPT) codes were used to identify prior parity and current contraceptive use **(Table 1)** .

**TABLE 1.** Description of Diagnostic Criteria for Outcomes of Interest for Endometriosis, U.S. Active Component Service Women, 2017–2024

Outcomes of Interest	ICD-9-CM	ICD-10-CM	CPT Codes	ICD-9-PCS	ICD-10-PCS	Pharmacological
Endometriosis	617 *	N80 *				
Menorrhagia	626.2, 626.3, 627.0	N92.0, N92.2, N92.4				
Polycystic ovarian syndrome (PCOS)	256.4	E28.2				
Uterine fibroids	218, 218.0, 218.1, 218.2, 218.9, 280.0 ^a^ , 285.1 ^a^ , 623.8 ^a^ , 626.2 ^a^ , 626.6 ^a^ , 626.5 ^a^ , 627.0 ^a^ , 626.8 ^a^ , 626.9 ^a^ , 625.0 ^a^ , 625.3 ^a^ , 625.5 ^a^ , 625.9 ^a^	D25, D25.0, D25.1, D25.2, D25.9, D50.0 ^a^ , D62 ^a^ , N89.8 ^a^ , N92.0 ^a^ , N92.1 ^a^ , N92.3 ^a^ , N92.4 ^a^ , N92.5 ^a^ , N92.6 ^a^ , N93.8 ^a^ , N93.9 ^a^ , N94.1 ^a^ , N94.4 ^a^ , N94.5 ^a^ , N94.6 ^a^ , N94.8 ^a^ , N94.89 ^a^ , N94.9 ^a^ , R10.2 ^a^				
Infertility	628, 628.0, 628.1, 628.2, 628.3, 628.4, 628.8, 628.9	N97, N97.0, N97.1, N97.2, N97.8, N97.9				
Parity	669.71, V27 * , 650 *	O82, O80, Z37 *				
Implant	V25.43, V25.5, V45.52	Z30.017, Z30.46	11975, 11981, 11983, 11977, J7307, J7306, 11976, 11982			Therapeutic drug class for contraceptive in implant form; prescription for Norplant
Injection		Z30.013, Z30.42	J1050, J1051, J1055, J1056			Prescription for Depo-Provera, Depo-SubQ Provera, or Medroxyprogesterone acetate in syringe or vial form
Intrauterine device (IUD)	V25.1, V25.11, V25.12, V25.13, V25.42, V45.51, 996.32	Z30.430, Z30.014, Z30.433, Z30.431, Z97.5, T83.31XA, T83.31XA, T83.31XD, T83.31XS, T83.32XA, T83.32XD, T83.32XS, T83.39XA, T83.39XD, T83.39XS, Z30.432	58300, J7302, J7296, J7297, J7298, J7301, J7300, 58301	697	0UH97HZ, 0UH-C7HZ, 0UHC8HZ, 0UH90HZ, 0UH-98HZ, 0UPD7HZ, 0UPD8HZ	Prescription for IUD; prescription for Mirena, Kyleena, Skyla, Liletta, or Paragard
Oral birth control		Z30.011, Z30.41				Therapeutic drug class for contraceptive in capsule or tablet form; prescription for Ortho-Novum or Norethindrone-ethinyl estradiol
Patch		Z30.016, Z30.45	J7304			Therapeutic class for contraceptive in patch form
Vaginal ring		Z30.015, Z30.44	J7303, J7295, J7294			Therapeutic class for contraceptive in vaginal ring form
Miscellaneous		Z30.018, Z30.019, Z30.02, Z30.09, Z30.40, Z30.49, Z30.8, Z30.9	96372, S4993, 57170			

Abbreviations: ICD-9-CM, International Classification of Diseases, 9th Revision, Clinical Modification; ICD-10-CM, International Classification of Diseases, 10th Revision, Clinical Modification; CPT, Current Procedural Terminology; ICD-9-PCS, International Classification of Diseases, 9th Revision, Procedure Coding System; ICD-10-PCS, International Classification of Diseases, 10th Revision, Procedure Coding System; PCOS, polycystic ovarian syndrome; IUD, intrauterine device.

* Indicates all codes following the parent code.

a Associated symptoms of uterine fibroids. Individuals can be diagnosed with uterine fibroids by a combination of association symptoms and uterine fibroid ICD codes.

Parity was defined as a delivery-related code **(Table 1)** in any diagnostic position prior to the incident date. Delivery events were counted once every 280 days and recorded as a binary variable (‘yes’ or ‘no’) and as a categorical variable representing the number of births (0–3+). Current contraceptive use was defined as use of at least 1 contraceptive type: implant, injection, IUD, oral birth control, patch, vaginal ring, or miscellaneous type (i.e., unspecified or not already listed). Service women were counted once per category. Current use for long-acting contraceptives was determined within 5 years preceding the incident date for IUDs and within 3 years preceding the incident date for implants; all other contraceptive types were determined as current use within 12 months preceding the incident date. Pharmaceutical data were also utilized to analyze contraceptive use for implants, injections, IUDs, oral birth control, patches, and vaginal rings **(Table 1)** .

### Case definition

A case of endometriosis was defined as an individual with 1 inpatient encounter with a case-defining code in any diagnostic position or 2 ambulatory encounters within 180 days with a case-defining code in any diagnostic position. ^8^ Individuals were counted as an incident case once per lifetime. ^8^

Menorrhagia was defined as an individual with 1 inpatient encounter with a case-defining code in the primary diagnostic position or 2 ambulatory encounters within 180-day period with a case-defining code in any diagnostic position. ^19^ Menorrhagia cases were counted once every 365 days. ^19^ PCOS was defined as an individual with 1 inpatient encounter with a case-defining code in the primary or secondary diagnostic position, or 2 ambulatory encounters in any diagnostic position. ^20^ Uterine fibroids were defined as 1 inpatient or ambulatory encounter with a case-defining code in the primary diagnostic position, or 1 inpatient or ambulatory encounter with a case-defining code in the secondary diagnostic position and at least 1 associated symptom **(Table 1)** in the primary diagnostic position. ^21^ Infertility was defined as 1 inpatient encounter with a case-defining code in the primary diagnostic position or 2 ambulatory encounters with a case-defining code in the primary or secondary diagnostic position. ^22^ PCOS and uterine fibroids counted once per lifetime, while infertility was counted once per surveillance period. ^20 - 22^

### Statistical analysis

Crude IRs for demographic variables and case year were calculated per 10,000 p-yrs. Parity and deployment were stratified by count, while contraceptive use was categorized by type, to assess trends. Incident rate ratios (IRRs) and 95% confidence intervals (CIs) were then calculated for baseline characteristics. Prior parity and deployment may include person-time occurring outside the surveillance period; therefore, overall person-time was used to calculate crude IRs. IRRs were not calculated for prior characteristics, as using the overall person-time produced non-comparable rate contrast. To estimate the health care burden of endometriosis, medical encounters with a case-defining code in the primary diagnostic position were examined to evaluate the total numbers of medical encounters, individuals affected, and hospital bed days. All analyses were conducted using SAS ^®^ Enterprise Guide ^®^ software (version 8.3, SAS Inst., Inc., Cary, NC).

## Results

During the 8-year surveillance period, 5,733 ACSW, or 1.3% of all eligible service women during the period, were diagnosed with an incident case of endometriosis, at an overall rate of 32.8 cases per 10,000 p-yrs **(Table 2)** .

**TABLE 2.** Baseline Characteristics, Incident Endometriosis Diagnoses, U.S. Active Component Service Women, 2017–2024

Baseline Characteristics	Total, 2017–2024					
	No.	Person-Years	Rate ^a^	IRR	95% LL	95% UL
Total	5,733	1,748,206.6	32.8	—	—	—
Age, *y*						
<20	101	141,070.9	7.2	1.0	Reference	Reference
20–24	1,188	577,167.7	20.6	2.9	2.4	3.5
25–29	1,261	431,240.9	29.2	4.1	3.3	5.0
30–34	1,165	276,482.7	42.1	5.9	4.8	7.2
35–39	1,071	186,552.6	57.4	8.0	6.5	9.8
40+	947	135,691.7	69.8	9.8	7.9	12.0
Race and ethnicity						
White, non-Hispanic	2,542	718,660.7	35.4	1.0	Reference	Reference
Black, non-Hispanic	1,673	423,604.0	39.5	1.1	1.1	1.2
Hispanic	880	355,801.2	24.7	0.7	0.7	0.8
Other	638	250,140.7	25.5	0.7	0.7	0.8
Military rank						
Enlisted	4,579	1,403,673.7	32.6	1.0	Reference	Reference
Officer, warrant officer	1,154	344,532.9	33.5	1.0	1.0	1.1
Branch of service						
Army	2,014	560,863.4	35.9	1.0	Reference	Reference
Navy	1,449	527,902.5	27.4	0.8	0.7	0.8
Marine Corps	228	128,365.0	17.8	0.5	0.4	0.6
Air Force, Space Force	2,042	531,075.7	38.5	1.1	1.0	1.1
Military occupation						
Infantry, artillery, combat engineering	119	51,450.7	23.1	1.0	Reference	Reference
Armor, motor transport	136	54,178.0	25.1	1.1	0.9	1.4
Pilot, air crew	70	28,658.7	24.4	1.1	0.8	1.4
Repair, engineering	958	340,316.8	28.2	1.2	1.0	1.5
Communications, intelligence	2,075	550,639.7	37.7	1.6	1.4	2.0
Health care	1,286	318,709.7	40.4	1.7	1.5	2.1
Other	1,089	404,253.0	26.9	1.2	1.0	1.4
Marital status						
Unmarried	1,651	800,034.0	20.6	1.0	Reference	Reference
Married	3,278	776,131.5	42.2	2.1	1.9	2.2
Other	804	172,041.2	46.7	2.3	2.1	2.5
Body mass index (BMI)						
Underweight (<18.5)	63	19,488.9	32.3	1.6	1.2	2.0
Normal (18.5–24.9)	2,077	793,643.1	26.2	1.0	Reference	Reference
Overweight (25–29.9)	2,348	682,481.5	34.4	1.3	1.2	1.4
Obese (30+)	1,238	240,217.3	51.5	2.0	1.8	2.1
Unknown	7	12,375.8	5.7	0.2	0.1	0.5

Abbreviations: IRR, incidence rate ratio; CI, confidence interval; LL, lower limit; UL, upper limit; *y* , years; BMI, body mass index.

a Rate per 10,000 person-years.

Overall, non-Hispanic Black women (IRR 1.1, 95% CI 1.1, 1.2) and women in health care occupations (IRR 1.7, 95% CI 1.5, 2.1) **(Table 2)** were more likely to be diagnosed with incident endometriosis than their counterparts. Additionally, women with a marital status of married (IRR 2.1, 95% CI 1.9, 2.2) or other (IRR 2.3, 95% CI 2.1, 2.5) were twice as likely to be diagnosed with incident endometriosis.

Rates of incident endometriosis increased with age, with women ages 40 years or older demonstrating the highest IR overall (69.8 cases per 10,000 p-yrs) **(Table 2)** . This trend was generally observed throughout the surveillance period (data not shown). Overall, compared to ACSW of normal BMI, overweight women were 31.0% more likely to be diagnosed with incident endometriosis, while underweight women were 57.0% more likely, and obese women were 97.0% more likely to be diagnosed with endometriosis **(Table 2)** .

Among women with incident endometriosis, 23.1% had co-occurring menorrhagia, while 21.1% had co-occurring infertility, 9.8% had co-occurring uterine fibroids, and 7.3 had co-occurring PCOS (data not shown).

Endometriosis cases with no prior deployments had higher IRs (19.1 cases per 10,000 p-yrs) compared to women with prior deployments (13.7 cases per 10,000 p-yrs) **(Table 3)** . Among women with prior deployments, women with 1 deployment had the highest IR (6.3 cases per 10,000 p-yrs) **(Table 3)** . On average, prior deployments lasted approximately 6 months and occurred about 9 years prior to the incident endometriosis diagnosis **(Table 3)** . Additionally, nulliparous women had a higher IR (22.3 cases per 10,000 p-yrs) compared to uniparous and multiparous women (10.5 cases per 10,000 p-yrs) **(Table 3)** . With each delivery, IRs of endometriosis decreased **(Table 3)** .

**TABLE 3.** Prior Deployment and Parity, Incident Endometriosis Diagnoses, U.S. Active Component Service Women, 2017–2024

Prior Characteristics	Total, 2017–2024		
	No.	Person-Years	Rate ^a^
Prior parity			
No	3,890	1,748,206.6	22.3
Yes	1,843	1,748,206.6	10.5
Prior parity, *n*			
0	3,890	1,748,206.6	22.3
1	905	1,748,206.6	5.2
2	678	1,748,206.6	3.9
Prior deployment			
No	3,332	1,748,206.6	19.1
Yes	2,401	1,748,206.6	13.7
Prior deployment, *n*			
0	3,332	1,748,206.6	19.1
1	1,094	1,748,206.6	6.3
2	597	1,748,206.6	3.4
3+	710	1,748,206.6	4.1

Average duration of deployment: 178 days

Average duration from deployment to incident endometriosis diagnosis: 8.9 years

Abbreviation: No., number; *n* , number.

a Rate per 10,000 person-years.

Among women with incident endometriosis, 24.0% were not currently using any form of contraceptive, while 76.0% were currently using some form of contraceptive **(Figure 2)** . Oral birth control was the most common (23.1%) type of contraceptive used by ACSW, followed by miscellaneous type (22.2%) and IUDs (16.7%) **(Figure 2)** .

**FIGURE 1.**
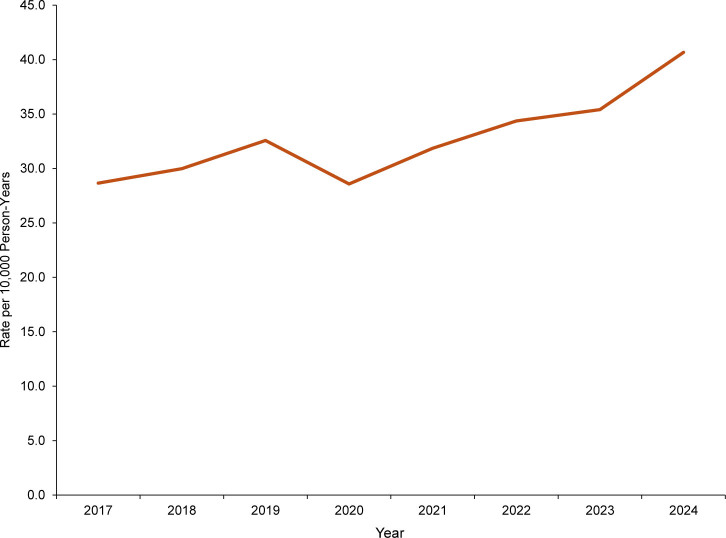
Incidence of Endometriosis, U.S. Active Component Service Women, 2017–2024

**FIGURE 2.**
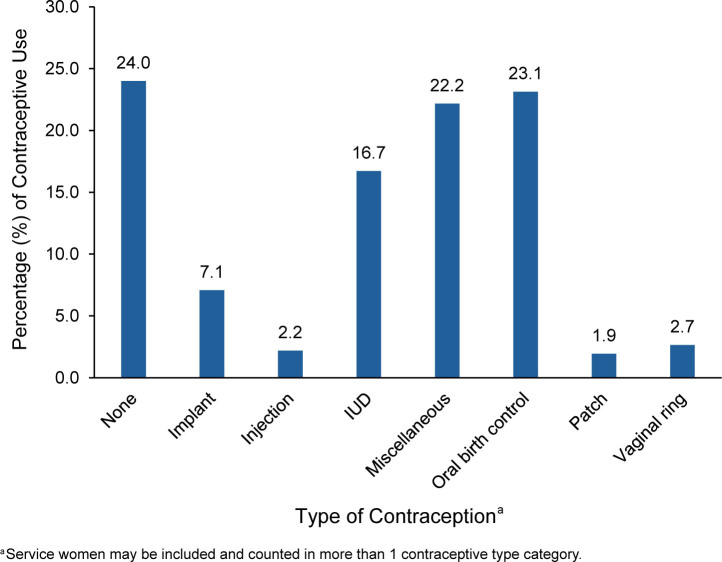
Percentage of Current Contraceptive Use, Incident Endometriosis Diagnoses, U.S. Active Component Service Women, 2017–2024

**FIGURE 3.**
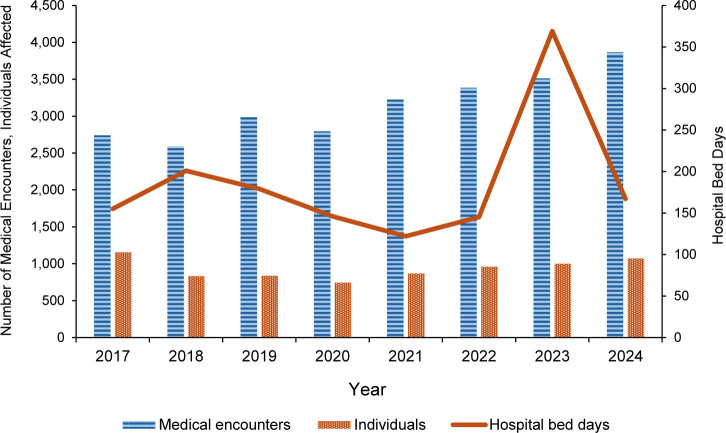
Burden of Endometriosis, U.S. Active Component Service Women, 2017–2024

**Figure 3** presents the burden of endometriosis among ACSW. The majority of 2017–2024 medical encounters for endometriosis were ambulatory care encounters **(Figure 3)** . The number of medical encounters remained relatively stable before 2021, when a continued annual increase began **(Figure 3)** . The number of individuals with medical encounters decreased in 2018, but since 2020 counts have increased, along with medical encounters **(Figure 3)** . The number of hospital bed days was at its lowest in 2021, while only 2 years later, in 2023, the highest number of hospital bed days was recorded. Counts more than doubled in 2023 compared to the previous year, but this is attributed to a small number of individuals rather than a reflection of the entire population **(Figure 3)** .

## Discussion

This study analyzed incidence of diagnosis rates of endometriosis, and this report describes the distributions of prior deployment, parity, and BMI on IRs. Co-occurring gynecological conditions, current contraceptive use, and health care burden were examined as well. Compared to the prior *MSMR* report on endometriosis, ^8^ overall crude incidence of endometriosis has increased from 30.8 cases per 10,000 p-yrs during 2012-2016 to 32.8 cases per 10,000 p-yrs in 2017-2024. During the surveillance period, IRs increased nearly 42.0% from 2017 to 2024. The current findings suggest an upward trend of newly diagnosed endometriosis among ACSW. When compared to civilian women, ACSW have greater accessibility to health care and diagnosis, provided by MHS, which may explain the increase in endometriosis diagnosis, rather than reflect a true increase in cases.

Consistent with the prior reporting of endometriosis among ACSW, ^8^ service women who were older, non-Hispanic Black race or ethnicity, and in health care occupations had higher rates of endometriosis; similar findings for age at diagnosis were reported for the general population. ^11^ Civilian women ages 36-45 years were found to have higher IRs of endometriosis. ^11^ Delayed diagnosis from symptom onset could explain why IRs are higher among women ages 35 years or older compared to younger service women.

Differences between racial and ethnic groups appear to be unique to the military population, when compared to the general population. Several studies have found lower incident endometriosis among non-Hispanic Black women compared to non-Hispanic White women, or no significant difference at all. ^11 , 23 , 24^ Disparities in civilian health care and need for surgical diagnosis may explain differences among military and civilian rates. ^23^ Further exploration of higher IRs of endometriosis among non-Hispanic Black women compared to civilian populations may be warranted.

Service women with no deployment history prior to endometriosis diagnosis had higher IRs of endometriosis compared to those with prior deployments, while women with 1 deployment had higher IRs than women with multiple deployments. These findings are somewhat unexpected, given the epidemiological associations between deployment and adverse reproductive and mental health outcomes. ^9 , 15^ The IRs are crude, however, and therefore the observed difference may be due to unadjusted confounding variables rather than a true statistical difference in risk. Combat trauma could negatively influence mental health, leading to riskier sexual behaviors and avoidance of reproductive health care ^11^ ; these factors lead to poorer reproductive health outcomes. ^13^

Determining the effects of deployment on endometriosis are difficult, however, due to the timing of disease onset and disease diagnosis. In this study, deployment occurred approximately 9 years, on average, prior to endometriosis diagnosis. Additionally, women with endometriosis demonstrate poor physical performance compared to women without the condition, ^25^ which may disqualify women from deployment, possibly inferring the ‘healthy warrior effect’, with healthier ACSW more likely to deploy. Furthermore, less than 4% of incident cases deployed following diagnosis (data not shown). These findings suggest that endometriosis diagnosis may inhibit deployment of ACSW and require greater medical management of ACSW to maintain force readiness.

BMI is reported to have an inverse relationship with endometriosis, ^2 , 6^ and in this study underweight women did have overall higher crude IRs than overweight women; this finding was not evidenced throughout the surveillance period, however (data not shown). Obese service women were observed to have the highest incidence of endometriosis overall, and throughout the study. Previous research found that this inverse relationship of BMI and endometriosis was not evident among women ages 30 years or older. ^26^ When this study examined BMI by age group, underweight ACSW ages 25-34 years had the highest IRs, while in all other age groups, obese ACSW had the highest rates (data not shown). These findings support the previous research, ^26^ suggesting a greater association of underweight BMI with younger ACSW and endometriosis diagnosis.

Lower parity was associated with higher rates of endometriosis. Given the association between infertility and endometriosis, ^17^ these findings may be unsurprising, however. In this population, 21.1% of women with incident endometriosis also had co-occurring infertility. Additionally, the majority of endometriosis cases in this population were currently using contraceptives prior to diagnosis. Pregnancy prevention is not the only indication for contraceptive use, as oral contraceptives are a primary or ‘first-line’ treatment for endometriosis and endometriosis-associated symptoms. ^27^ Oral birth control was the most common contraceptive type among women in this population.

Several limitations are important to consider when interpreting these findings. The delay in obtaining endometriosis diagnosis ^5^ creates a challenge for determining disease onset and how reproductive health, demographic, and service-related factors affect the condition. The IRs for prior parity and prior deployment were calculated using overall person-time due to an inability to calculate person-time prior to diagnosis for the population at risk, as not all ACSW at risk in the population were diagnosed with endometriosis. Additionally, the ‘healthy warrior effect’ may explain higher IRs among ACSW with no deployment history compared to those with deployment history. The crude IRs for prior parity and prior deployment should be evaluated as preliminary and with caution. Lack of a standardized case definition for endometriosis in the literature presents comparison challenges. ^3 , 4^ Additionally, the difficulty of obtaining a diagnosis may obscure true incidence. Furthermore, results related to parity and contraceptive use are observational. Reasons why women are nulliparous or contraceptive users are numerous and unknown in this study. Finally, deployment data were only available through 2022, under-estimating rates of prior deployment among ACSW.

Endometriosis is associated with a multitude of symptoms ^2^ that can affect military readiness and quality of life. Future studies should evaluate endometriosis severity to understand its effects on force readiness and health care provision. A more comprehensive cohort study of symptomology, mental health, deployment, and demographics, from accession to end-of-service contract, may better explain the effects of military service on endometriosis.
